# Early Recognition and Management of Complex Regional Pain Syndrome Following Distal Radius Fracture: A Structured Narrative Review

**DOI:** 10.7759/cureus.110714

**Published:** 2026-06-12

**Authors:** Glenn Briffa, Mark-Anthony Scicluna, Matthew Spiteri, Neill Zammit, Jordan Calleja, Simon Aquilina

**Affiliations:** 1 Surgery, Mater Dei Hospital, Msida, MLT; 2 Medicine, Mater Dei Hospital, Msida, MLT; 3 Internal Medicine, Mater Dei Hospital, Msida, MLT; 4 Neurosurgery, Mater Dei Hospital, Msida, MLT; 5 General Surgery, Mater Dei Hospital, Msida, MLT; 6 Orthopedics and Trauma, Mater Dei Hospital, Msida, MLT

**Keywords:** complex regional pain syndrome, crps, distal radius fracture, early diagnosis, early mobilisation, pain sensitisation, rehabilitation, wrist fracture

## Abstract

Complex regional pain syndrome (CRPS) is a chronic pain disorder that may develop following a distal radius fracture and can result in significant functional impairment and prolonged disability. Early diagnosis remains challenging because symptoms frequently overlap with normal post-fracture recovery. This structured narrative review evaluated current evidence regarding the timing of diagnosis, early predictors, and early management strategies influencing outcomes of CRPS following distal radius fracture. A structured literature search of PubMed/MEDLINE and Embase was performed for English-language studies published between 2000 and 2026 using combinations of keywords related to CRPS, distal radius fracture, diagnosis, risk factors, prevention, and management. Studies involving adult patients with CRPS following distal radius or wrist fracture and addressing diagnosis, predictors, or early management strategies were included. Owing to heterogeneity in study designs and outcome measures, findings were synthesized using thematic narrative analysis. Twenty-six studies met the inclusion criteria. Evidence suggests that clinical and physiological abnormalities associated with CRPS may emerge during the early post-injury period, although formal diagnosis is often established weeks to months after fracture treatment. Higher early pain intensity, enhanced pain sensitization, fibromyalgia, female sex, older age, lower vitamin D levels, and certain genetic polymorphisms were associated with an increased risk of CRPS. Early mobilization and structured rehabilitation strategies were associated with improved functional outcomes and lower reported CRPS incidence in several studies. Evidence regarding vitamin C prophylaxis was heterogeneous, with meta-analyses suggesting potential benefit while individual randomized controlled trials reported inconsistent findings. Imaging modalities demonstrated limited sensitivity for early diagnosis, reinforcing the importance of longitudinal clinical assessment. CRPS remains an uncommon but clinically important complication following a distal radius fracture. Current evidence supports the concept that altered recovery trajectories and early pain amplification may precede formal diagnosis. Careful monitoring of recovery, recognition of disproportionate symptoms, and promotion of early functional rehabilitation may facilitate earlier identification and management. Further prospective studies using standardized diagnostic criteria are required to better define early predictors and optimize preventive strategies.

## Introduction and background

Complex regional pain syndrome (CRPS) is a chronic pain condition characterized by regional pain disproportionate to the inciting event and accompanied by sensory, vasomotor, sudomotor, and motor or trophic disturbances [1]. The syndrome most commonly develops following trauma or surgery and is associated with substantial functional impairment, reduced quality of life, and prolonged disability [2]. Diagnosis remains clinical and relies on established diagnostic criteria integrating symptoms and observable signs across multiple physiological domains, reflecting the absence of a definitive diagnostic test [1].

Current understanding recognizes CRPS as a multifactorial disorder involving peripheral inflammation, autonomic dysregulation, and central sensitization, with interactions between peripheral and central nervous system mechanisms contributing to symptom persistence [3,2]. Despite advances in the understanding of its pathophysiology, clinical outcomes remain variable, and many patients experience chronic pain and functional limitation [4].

Orthopedic trauma represents one of the most common precipitating contexts for CRPS, with distal radius fractures frequently identified as a principal trigger. Prospective epidemiological studies demonstrate that CRPS develops in a measurable proportion of patients following fractures, confirming its relevance within routine orthopedic practice. In a large multicentre prospective cohort study of patients presenting with wrist fractures, approximately 3.8% developed CRPS within four months of injury, highlighting the clinical significance of this complication even after standard fracture management [5].

Importantly, early post-injury clinical characteristics appear predictive, with higher pain intensity during the first week after fracture strongly associated with subsequent CRPS development. These findings suggest that early disease trajectories may be identifiable during routine fracture follow-up [5].

A major clinical challenge lies in distinguishing early CRPS from expected post-traumatic recovery. Pain, swelling, stiffness, and reduced movement commonly occur after a fracture and may obscure early pathological changes, contributing to diagnostic delay. As CRPS lacks confirmatory investigations and diagnosis depends primarily on clinical assessment, recognition is frequently postponed until functional impairment becomes established [3,4]. Evidence increasingly supports early multidisciplinary management and restoration of limb function as key determinants of recovery, emphasizing the importance of timely identification within orthopedic pathways [2].

Although extensive literature describes CRPS mechanisms and treatment strategies, relatively few reviews synthesize evidence specifically within the context of orthopedic trauma care, particularly regarding diagnostic timing and early management following distal radius fractures. Given the frequency of these injuries and the potential for preventable long-term disability, integration of CRPS recognition into fracture clinic practice may therefore be important for improving early identification and management.

This structured narrative review, therefore, examines the timing of diagnosis, predictors of disease development, and early management strategies influencing outcomes of complex regional pain syndrome following distal radius fractures, with emphasis on implications for orthopedic clinical practice.

## Review

Methodology

This study was conducted as a structured narrative review guided by principles for rigorous narrative synthesis as described in the Scale for the Assessment of Narrative Review Articles (SANRA) [6]. The methodological approach aimed to achieve transparency and reproducibility consistent with systematized literature reviews while acknowledging that the present study was not designed as a formal systematic review or PRISMA-guided meta-analysis.

The review question was defined a priori as follows: What is known about the timing of diagnosis, early predictors, and early management strategies influencing outcomes of complex regional pain syndrome following distal radius fractures?

A literature search was performed using the PubMed/MEDLINE and Embase databases to identify relevant studies addressing this question. The search strategy included combinations of keywords and indexed terms related to complex regional pain syndrome (CRPS), distal radius fracture, wrist fracture, early diagnosis, risk factors, and prevention, as indicated in Table 1 below. Filters were applied to include human studies published in English between 2000 and 2026 to capture contemporary literature reflecting current diagnostic criteria and management practices.

**Table 1:** Literature search strategy used for study identification CRPS - complex regional pain syndrome

Database	Search Terms	Filters applied	Records Identified
PubMed/MEDLINE	("complex regional pain syndrome" OR CRPS) AND ("distal radius fracture" OR "wrist fracture") AND ("early diagnosis" OR diagnosis OR predictors OR "risk factors" OR prevention OR management OR rehabilitation)	Humans, English language, 2000–2026	84
Embase	("complex regional pain syndrome" OR CRPS) AND ("distal radius fracture" OR "wrist fracture") AND ("early diagnosis" OR diagnosis OR predictors OR "risk factors" OR prevention OR management OR rehabilitation)	Humans, English language, 2000–2026, MEDLINE overlap excluded	174

The PubMed/MEDLINE search yielded 84 records. Following title screening for relevance, 33 articles were retained for abstract review. A complementary search was subsequently conducted in Embase with MEDLINE overlap excluded to minimize duplication. This search identified 174 records, of which 25 studies remained following title screening and proceeded to abstract assessment. In total, 58 studies underwent abstract screening.

Study selection was performed in two stages. Titles were initially screened to identify studies relevant to complex regional pain syndrome occurring after a distal radius fracture. Abstracts of potentially eligible articles were then reviewed against predefined inclusion and exclusion criteria. Following abstract review, removal of duplicates, and exclusion of non-relevant studies, a total of 26 articles were included in the final qualitative synthesis.

Studies were eligible for inclusion if they involved adult patients sustaining distal radius or wrist fractures and evaluated complex regional pain syndrome arising following injury. Inclusion and exclusion criteria are depicted in Table 2 below. Included studies addressed at least one of the following domains: timing or early diagnosis of CRPS, predictors or risk factors associated with its development, or early management and preventive strategies influencing clinical outcomes. Original clinical investigations, randomized controlled trials, cohort studies, observational studies, and relevant systematic reviews were considered eligible, provided they were published in English between 2000 and 2026, and full texts were accessible.

**Table 2:** Inclusion and exclusion criteria CRPS - complex regional pain syndrome

Inclusion criteria	Exclusion criteria
Adult patients (≥18 years) with distal radius fracture or wrist fracture	Studies involving CRPS unrelated to distal radius or wrist fracture
Studies evaluating CRPS occurring following distal radius or wrist fracture	Studies focused exclusively on chronic, refractory, or established CRPS without a fracture-specific context
Studies addressing timing of diagnosis or early recognition of CRPS	Studies not reporting outcomes relevant to diagnosis, predictors, prevention, or early management
Studies evaluating predictors or risk factors for CRPS development	Editorials, letters, commentaries, and expert opinions without original data
Studies evaluating preventive or early management strategies	Conference abstracts without full published data
Original clinical investigations, cohort studies, observational studies, randomized controlled trials, and systematic reviews	Study protocols without outcome data
Human studies	Animal or laboratory studies
Full-text articles available	Articles with inaccessible full text
Published in English	Non-English publications
Published between 2000 and 2026	Publications before 2000

Studies were excluded if they investigated CRPS unrelated to distal radius fractures, focused exclusively on chronic or refractory CRPS populations without a fracture-specific context, or failed to report outcomes relevant to diagnosis, predictors, or early management. Editorials, conference abstracts, protocols without outcome data, errata, and non-English publications were also excluded.

Given the heterogeneity of study designs and outcome measures among included studies, findings were synthesized using a thematic narrative approach rather than quantitative meta-analysis. Evidence was organized into three predefined domains aligned with the review objective: timing of diagnosis and early recognition, predictors and risk factors, and early management and preventive strategies.

Results

Timing of Diagnosis and Early Recognition of CRPS Following Distal Radius Fracture

The timing of diagnosis of complex regional pain syndrome (CRPS) following distal radius fracture varied across studies and was influenced by diagnostic criteria, follow-up duration, and assessment methodology. Reported incidence and timing estimates differed substantially between prospective clinical studies and large administrative datasets, reflecting differences in case ascertainment and diagnostic thresholds. Studies pertaining to this are depicted in Table 3. 

**Table 3:** Studies examining the timing of diagnosis and early recognition of complex regional pain syndrome following distal radius fracture IASP - International Association for the Study of Pain; CRPS - complex regional pain syndrome

Author and year	Study design	Population	Key findings
Żyluk and Mosiejczuk (2013) [7]	Prospective cohort	Distal radius fracture surgery patients	CRPS incidence 8.3% at six weeks using modified IASP criteria
Schürmann et al. (2000) [8]	Physiological study	Distal radius fracture patients	Autonomic dysfunction detected in patients who later develop CRPS
Schürmann et al. (2007) [9]	Diagnostic study	Suspected CRPS after fracture	Thermography and bone scintigraphy identify early abnormalities
Melbye et al. (2026) [10]	Registry cohort	National fracture registry	CRPS occurs in a minority of distal radius fracture cases
Goris et al. (2007) [11]	Observational clinical study	Post-traumatic CRPS patients	Early disproportionate symptoms may precede confirmed diagnosis

Prospective clinical investigations applying diagnostic criteria during early follow-up have reported that manifestations compatible with CRPS may be detectable within the first weeks after fracture treatment. In a cohort study evaluating patients following surgical treatment of distal radius fractures, CRPS, defined according to modified International Association for the Study of Pain (IASP) criteria, was identified in approximately 8.3% of patients at six weeks postoperatively, with fewer cases present at twelve weeks as symptoms resolved in some individuals [7].

Physiological investigations have also identified early abnormalities in patients who subsequently develop CRPS. Studies examining peripheral sympathetic nervous system function after radial fracture demonstrated diminished sympathetic vasoconstrictor responses in patients who later developed CRPS, suggesting that autonomic disturbances may be present early in the disease process [8].

However, diagnostic uncertainty during early fracture recovery is frequently reported. Imaging-based investigations evaluating thermography, magnetic resonance imaging, and three-phase bone scintigraphy have demonstrated limited sensitivity for identifying early CRPS, with substantial overlap between imaging findings observed in CRPS and those seen during normal post-traumatic healing [9]. These findings emphasize that CRPS remains primarily a clinical diagnosis and that imaging modalities have limited value as screening tools during early post-traumatic recovery.

Population-level registry studies have reported substantially lower incidence rates of CRPS following distal radius fracture compared with prospective clinical cohorts [10]. These studies suggest that when diagnosis relies on coded clinical diagnoses rather than prospective clinical evaluation, CRPS is identified in only a small minority of fracture patients.

Across the included studies, formal diagnosis was most commonly established during follow-up several weeks to months after fracture treatment rather than immediately after injury, reflecting the clinical challenge of distinguishing early CRPS from normal inflammatory responses associated with fracture healing [11].

Predictors and Risk Factors for Development of CRPS Following Distal Radius Fracture

Studies investigating predictors of CRPS following distal radius fracture identified several domains of potential risk factors, including patient characteristics, early symptom trajectories, biological susceptibility, and treatment-related variables, as represented in Table 4.

**Table 4:** Studies examining predictors and risk factors associated with the development of complex regional pain syndrome following distal radius fracture CRPS - complex regional pain syndrome

Author and year	Study design	Population	Key findings
Moseley et al. (2014) [5]	Prospective multicentre cohort	Patients presenting with wrist fractures	Higher pain intensity during the first week after fracture strongly predicted later CRPS development
Melbye et al. (2026) [10]	Registry cohort study	National distal radius fracture registry	CRPS occurred in a small proportion of fracture patients overall
Crijns et al. (2018) [12]	Observational cohort	Distal radius fracture patients	Fibromyalgia associated with increased likelihood of developing CRPS
Lipman et al. (2019) [13]	Observational cohort	Distal radius fracture patients	Patients with fibromyalgia or chronic pain disorders had higher rates of CRPS
Xu et al. (2024) [14]	Multivariate cohort analysis	Patients undergoing distal radius fracture surgery	Early postoperative symptom progression independently predicted CRPS development
Kaya et al. (2025) [15]	Retrospective case–control	Nonoperatively treated distal radius fracture patients	Certain clinical and treatment characteristics were associated with CRPS occurrence
Lee et al. (2020) [16]	Prospective cohort	Postmenopausal women undergoing distal radius fracture fixation	Lower vitamin D levels were associated with increased CRPS incidence
Herlyn et al. (2010) [17]	Genetic association study	CRPS patients compared with fracture controls	Differences in cytokine and adrenergic receptor gene polymorphisms associated with CRPS susceptibility
Roh et al. (2019) [18]	Prospective cohort	Distal radius fracture patients undergoing rehabilitation	Enhanced pain responses during rehabilitation were associated with later CRPS diagnosis
Rai et al. (2024) [19]	Cohort study	Distal radius fracture patients	Differences in CRPS incidence observed between fracture management approaches

Patient-related susceptibility factors were frequently reported. Large administrative database analyses identified increased rates of CRPS diagnosis among patients with pre-existing fibromyalgia compared with fracture patients without chronic pain conditions [12,13]. These findings have been interpreted as supporting the hypothesis that individuals with underlying pain sensitization disorders may be more susceptible to exaggerated post-traumatic pain responses.

Demographic characteristics have also been associated with CRPS occurrence. Observational studies reported higher rates of CRPS among female patients and older individuals sustaining distal radius fractures [14,15].

Biological factors were also explored. One cohort study reported that lower serum vitamin D levels were associated with increased CRPS incidence among postmenopausal women undergoing surgical fixation of distal radius fractures [16]. In addition, genetic investigations analyzing polymorphisms in inflammatory and adrenergic receptor genes identified differences in allele frequencies between patients who developed CRPS and fracture control populations [17].

Early symptom trajectories were another area of investigation. Prospective cohort studies examining postoperative recovery patterns reported that higher pain intensity and greater functional disability during early follow-up were associated with subsequent CRPS diagnosis [5]. Studies assessing pain sensitization during early recovery similarly reported that enhanced pain responses were associated with later CRPS development [18].

Treatment-related factors were also examined. Observational studies reported associations between prolonged immobilization and increased CRPS occurrence following nonoperative management of distal radius fractures [15]. Other studies evaluating fracture characteristics and surgical variables suggested that fracture complexity and reduction techniques may influence CRPS occurrence, although findings were inconsistent [19].

Taken together, the available evidence suggests that the development of CRPS following distal radius fracture likely reflects a multifactorial interaction between early post-traumatic physiological responses and individual patient susceptibility rather than fracture characteristics alone.

Early Management and Preventive Strategies Following Distal Radius Fracture

A range of early management strategies has been investigated in relation to the prevention and early treatment of CRPS following distal radius fracture. These include pharmacological prophylaxis, rehabilitation approaches, and service-level clinical interventions as depicted in Table 5. 

**Table 5:** Studies evaluating early management and preventive strategies for complex regional pain syndrome following distal radius fracture CRPS - complex regional pain syndrome

Author and year	Study design	Population	Key findings
Rai et al. (2024) [19]	Cohort study	Distal radius fracture patients	Different treatment approaches associated with variation in CRPS occurrence
Meena et al. (2015) [20]	Meta-analysis	Distal radius fracture studies	Vitamin C associated with lower CRPS risk after distal radius fracture
Aïm et al. (2017) [21]	Systematic review and meta-analysis	Studies of wrist fractures	Vitamin C administration associated with reduced CRPS incidence following wrist fracture
Ekrol et al. (2014) [22]	Randomized controlled trial	Patients with distal radial fractures	No statistically significant reduction in CRPS incidence compared with placebo
Malay & Chung (2014) [23]	Secondary analysis / review	Studies of CRPS prevention	Highlighted variability in CRPS diagnostic criteria and outcome definitions across trials
Alimian et al. (2021) [24]	Randomized controlled trial	Patients undergoing distal radius fracture surgery	Regional vitamin C administration associated with lower CRPS incidence
Boersma et al. (2022) [25]	Prospective cohort	Distal radius fracture patients	Early activity associated with lower CRPS incidence compared with prolonged immobilisation
Gutiérrez-Espinoza et al. (2022) [26]	Prospective rehabilitation study	Elderly patients with CRPS following distal radius fracture	Rehabilitation associated with improved functional outcomes
Cowell et al. (2018) [27]	Service evaluation	Hand therapy service patients	Service-level intervention associated with reduced CRPS incidence

Pharmacological prevention has most frequently been investigated in relation to vitamin C supplementation. Systematic reviews and meta-analyses have reported reduced CRPS incidence among patients receiving vitamin C following wrist fracture compared with control groups [20,21]. However, individual randomized trials have produced heterogeneous findings, with some studies failing to demonstrate a statistically significant reduction in CRPS incidence [22]. Methodological variation across studies, including differences in diagnostic criteria and outcome definitions, has been highlighted as a potential explanation for these inconsistent findings [23].

Regional administration of vitamin C as an adjunct to Bier block anesthesia during surgical management of distal radius fractures has also been evaluated. In a randomized controlled trial, patients receiving regional vitamin C demonstrated a lower incidence of CRPS compared with control participants [24].

Rehabilitation strategies have also been explored. Prospective studies examining early mobilization protocols reported lower rates of CRPS among patients encouraged to resume early activity following fracture treatment compared with those managed with prolonged immobilization [25]. Structured physiotherapy programs in patients with established CRPS following distal radius fracture have also been associated with improvements in pain and functional outcomes during follow-up [26].

Service-level interventions aimed at improving early recognition have also been reported. Implementation of structured clinical pathways and educational interventions within hand therapy services has been associated with reductions in reported CRPS incidence among distal radius fracture patients [27].

Across the included studies, early management strategies were evaluated using heterogeneous study designs, including randomized controlled trials, cohort studies, and observational implementation studies. Consequently, while several interventions appear promising, the overall evidence base remains limited by variation in diagnostic criteria, outcome definitions, and methodological quality.

Discussion

This review synthesized evidence examining the timing of diagnosis, predictors of disease development, and early management strategies associated with complex regional pain syndrome (CRPS) following distal radius fractures. Across the included studies, several consistent themes emerge. Evidence suggests that clinical and physiological changes associated with CRPS may arise during the early post-injury recovery period, while formal diagnosis is often established later in the clinical course. At the same time, the literature indicates that disease development is influenced by both early symptom trajectories and individual patient susceptibility rather than fracture characteristics alone. These observations provide insight into how normal recovery following distal radius fracture may diverge toward persistent pain syndromes in a small subset of patients.

CRPS as an Early Evolving Process Following Distal Radius Fracture

The findings of this review support the concept that CRPS following distal radius fracture may represent an evolving pathological process that begins during the early stages of recovery rather than a complication that emerges abruptly later in the healing course. Collectively, the included studies suggest that physiological disturbances may precede the point at which patients fulfill established diagnostic criteria for CRPS.

Evidence of early autonomic dysfunction and abnormalities identified on adjunctive investigations such as thermography and bone scintigraphy support the presence of biological changes during the initial post-injury period, although the diagnostic utility of these modalities remains limited [8,9]. This reinforces the view that CRPS is primarily a clinical diagnosis and that ancillary investigations should be interpreted cautiously.

An important challenge is the overlap between early CRPS manifestations and expected post-fracture recovery. Pain, edema, stiffness, and reduced mobility are common after a distal radius fracture, making it difficult to distinguish normal healing from the onset of pathological processes. Consequently, formal diagnosis is often delayed until symptoms persist or recovery deviates from the anticipated trajectory, despite evidence that atypical features may be apparent within the first weeks after injury [7,11].

Although these observations support heightened awareness during early follow-up, population-based data demonstrate that CRPS remains an uncommon outcome after distal radius fracture [10]. This suggests that early post-traumatic changes alone are insufficient for disease development and that progression to persistent CRPS is likely influenced by additional patient-specific susceptibility factors.

Interaction Between Injury Response and Patient Susceptibility

The available evidence also suggests that CRPS development reflects an interaction between the biological response to tissue injury and individual patient susceptibility. Although distal radius fractures are common orthopedic injuries, only a minority of patients develop CRPS during recovery [10], indicating that the injury itself is unlikely to be sufficient to explain disease emergence.

Several studies highlight the importance of early symptom trajectories in identifying patients at increased risk. Prospective cohort investigations demonstrated that higher pain intensity during the first week following wrist fracture was strongly associated with subsequent CRPS diagnosis [5]. Similarly, studies assessing pain sensitization during early rehabilitation reported exaggerated pain responses among patients who later developed CRPS [18]. These findings suggest that early amplification of nociceptive processing may represent a key step in the transition from acute post-traumatic pain to persistent pain syndromes.

Patient-related factors also appear to contribute to disease susceptibility. Observational studies reported higher CRPS rates among patients with pre-existing fibromyalgia compared with fracture patients without chronic pain conditions [12,13]. These findings support the hypothesis that individuals with underlying central sensitization disorders may be predisposed to exaggerated post-traumatic pain responses.

Biological susceptibility has also been explored. Lower serum vitamin D levels were associated with increased CRPS incidence in postmenopausal women undergoing surgical fixation of distal radius fractures [16]. In addition, genetic investigations have identified polymorphisms in adrenergic receptor pathways associated with CRPS development following distal radius fracture [17]. While these findings remain preliminary, they suggest that genetic and metabolic factors may contribute to individual vulnerability.

In contrast, evidence relating to fracture management variables appears less consistent. Some studies reported associations between treatment characteristics and CRPS occurrence [19,15], but these findings were not uniformly observed and may reflect confounding factors such as fracture severity or immobilization duration.

Taken together, the literature supports a multifactorial model in which distal radius fracture provides the initiating stimulus, while variability in nociceptive processing, inflammatory regulation, and autonomic function determines whether recovery proceeds normally or progresses toward persistent pain syndromes.

Implications for Early Identification and Orthopedic Practice

The findings of this review have several implications for clinical management following a distal radius fracture. Across multiple studies, early clinical features-including disproportionate pain intensity and altered pain responses-were consistently associated with later CRPS diagnosis [5,18]. These observations suggest that routine clinical assessment during early follow-up may help identify patients demonstrating atypical recovery trajectories.

Diagnostic uncertainty during early recovery remains a key challenge. Studies evaluating diagnostic modalities demonstrated limited sensitivity of imaging techniques and highlighted the importance of longitudinal clinical assessment in identifying early CRPS [9]. As a result, careful monitoring of symptom progression during fracture follow-up may be more informative than reliance on a single time-point diagnostic evaluation.

Evidence relating to rehabilitation strategies further underscores the importance of early functional recovery. Prospective evaluation of post-injury activity protocols demonstrated lower CRPS rates among patients encouraged to resume early activity compared with those managed with prolonged immobilization [25]. Structured physiotherapy interventions have also been associated with improved functional outcomes in patients with established CRPS following distal radius fracture [26].

Pharmacological prevention strategies have produced heterogeneous findings. Meta-analyses reported reduced CRPS incidence with vitamin C supplementation following wrist fracture [20,21], whereas randomized controlled trial data did not consistently demonstrate statistically significant benefit [22]. Additional investigation of regional vitamin C administration reported lower CRPS incidence in a surgical cohort [24]. Variation in diagnostic criteria and study design likely contributes to these inconsistent findings.

Limitations

This review has several limitations that should be considered when interpreting its findings. First, the study was conducted as a structured narrative review rather than a formal systematic review; therefore, study selection and synthesis were not performed using the PRISMA methodology or formal risk-of-bias assessment tools. Although database searches were conducted systematically across PubMed/MEDLINE and Embase using predefined eligibility criteria, the narrative design introduces the possibility of selection bias.

Second, substantial heterogeneity existed among the included studies with respect to diagnostic criteria for CRPS, study populations, treatment approaches, and follow-up durations. Variability in diagnostic frameworks, including differing applications of IASP-based and Budapest criteria, may have influenced reported incidence rates and the timing of diagnosis across studies.

Third, many of the included studies employed observational designs, including retrospective database analyses and cohort studies. These study designs limit causal inference and may be subject to confounding factors such as fracture severity, immobilization duration, and differences in treatment protocols.

Finally, differences in outcome definitions and reporting methods across studies limited the ability to directly compare findings or perform quantitative synthesis. As a result, conclusions regarding predictors and preventive strategies should be interpreted cautiously. Despite these limitations, consistent patterns were identified across diverse study designs, supporting the thematic synthesis presented in this review.

## Conclusions

Complex regional pain syndrome remains an uncommon but clinically important complication following a distal radius fracture. Evidence reviewed in this study suggests that early alterations in pain processing and recovery patterns may precede formal diagnosis, while individual patient susceptibility appears to influence progression from normal healing to persistent pain syndromes.

Overall, the available evidence indicates that early identification of atypical recovery patterns, promotion of early mobilization, and timely referral for structured rehabilitation are important components of clinical management following distal radius fracture. Careful monitoring of patients demonstrating disproportionate pain or delayed functional improvement may facilitate earlier recognition of CRPS and improve opportunities for timely intervention.
